# Gonorrhœa

**Published:** 1852-02

**Authors:** 


### RECORD OF MEDICAL SCIENCE.

### SURGERY.

*Gonorrhœa.* Dr. C. B. Gibson, (Medical Society of Virginia, November meeting,) made some remarks on the treatment of *Gonorrhœa,* and exhibited an instrument which he learned was in common use in New York, and which he thought would be found useful in cases where it was desirable to apply caustic solution to every part of the urethra. The instrument consists of a silver tube, containing a sliding wire, which is rolled at its end with fine sponge. The sponge may be saturated with any solution, and made to swab out the urethra and touch its entire wall. The instrument was introduced, he believed, by Dr. Campbell Stuart of New York, and he learned that it was very popular.

Dr. Gooch said that the instrument could be obtained from several of the druggists of the city, but that he did not like its construction nor its use. Since the subject was brought up, he would take the occasion to remark, that, having experienced great difficulty in the treatment of this

*opprobrium* of surgery, as Dr. Gibson had justly called gonorrhœa, he had lately given a fair trial to the *hydrastis canadensis* in five cases. He was sorry to report, however, that he had failed in accomplishing *any good whatever* with it. It is an astringent, but has no peculiar virtues, he thought. He was induced to make a trial of it many months ago, by the recommendation of a member of the society, who knew it as an old Philadelphia remedy. Just now, however, almost every medical journal in the United States is copying a short article by Dr. McCann of Ohio, in which he lays claim to originality in its use in this disease. He thought the claim as absurd as the specific.—*Stethoscope.*

